# Validation of an ion selective electrode system for the analysis of serum fluoride ion

**DOI:** 10.1155/S1463924695000344

**Published:** 1995

**Authors:** Ellie B. Duly, Stephen R. Luney, Thomas R. Trinick, James M. Murray, John E. A. Comer

**Affiliations:** 1 Department of Clinical Chemistry The Ulster Hospital Belfast BT16 0RH UK; 2 Department of Anaesthetics The Ulster Hospital Belfast BT16 0RH UK; 3 Orion Research (UK) East Sussex RH18 5ES UK; 4 14 Huntingdale Ballyclare BT39 9XB UK

## Abstract

A high impedance unit was developed for use with a fluoride/pH electrode system for the measurement of serum fluoride. The linearity, accuracy, precision and detection limit of the system is reported. At a pH of 1.55, the system was linear over a range of serum fluoride concentrations up to 100 μmol l^-1^, with a lower limit of detection of 0.3 μmol l^-1^. Recoveries at this pH were 94-105% in the range 2.6-100 μmol l^-1^. Within-run CVs ranged from 4.2% at a level of 2.3 μmol l^-1^ to 1.2% at a level of 55.7 μmol l^-1^, while day-to-day CVs ranged from 12.8% at a level of 2.2 μmol l^-1^ to 4.6% at a level of 51.7 μmol l^-1^. The system demonstrated a rapid response time and has the potential for a smaller sample size requirement with alternative electrode shape. Continued development of this unit into an automated fluoride ion selective electrode system is recommended, since the measurement of serial serum fluoride samples is of greatest importance in assessing the impact of new anaesthetic agents on renal function.


Journal of Automatic Chemistry, Vol. 17, No. 6 (November–December 1995), pp. 219–223

### Validation of an ion selective electrode system for the analysis of serum fluoride ion

**Ellie B. Duly[1], Stephen R. Luney[2], Thomas R. Trinick[1], James M. Murray[2] and John E. A. Comer[3]**

*1. Department of Clinical Chemistry, The Ulster Hospital, Belfast BT16 0RH, UK*
*2. Department of Anaesthetics, The Ulster Hospital, Belfast BT16 0RH, UK*
*3. Orion Research (UK), East Sussex RH18 5ES, UK*

*A high impedance unit was developed for use with a fluoride/pH electrode system for the measurement of serum fluoride. The linearity, accuracy, precision and detection limit of the system is reported. At a pH of 1·55, the system was linear over a range of serum fluoride concentrations up to 100 μmol $l^{-1}$, with a lower limit of detection of 0·3 μmol $l^{-1}$. Recoveries at this pH were 94–105% in the range 2·6–100 μmol $l^{-1}$. Within-run CVs ranged from 4·2% at a level of 2·3 μmol $l^{-1}$ to 1·2% at a level of 55·7 μmol $l^{-1}$, while day-to-day CVs ranged from 12·8% at a level of 2·2 μmol $l^{-1}$ to 4·6% at a level of 51·7 μmol $l^{-1}$. The system demonstrated a rapid response time and has the potential for a smaller sample size requirement with alternative electrode shape. Continued development of this unit into an automated fluoride ion selective electrode system is recommended, since the measurement of serial serum fluoride samples is of greatest importance in assessing the impact of new anaesthetic agents on renal function.*

### Introduction

Inorganic fluoride is known to produce a vasopressin-resistant, high output renal failure by its effect on the renal tubular cells of the ascending limb of the loop of Henle [1–4]. A number of new volatile anaesthetic agents currently under evaluation for future clinical use are heavily fluorinated, and undergo dehalogenation to free fluoride ion [5–7]. As a result, there has been renewed interest in the rapid and accurate measurement of fluoride ion in serum. The fluoride ion selective electrode is conventionally used to measure fluoride ion activity in aqueous solutions which have been buffered to about pH 5·5. At this pH, inorganic fluoride is fully ionized. The fluoride electrode potential is then measured against a reference half-cell with a liquid junction, such as a silver/silver chloride reference electrode.

The lower limit for linear Nernstian response of the fluoride electrode at pH 5·5 is typically about 10 μM [8]. It is known that ion-selective electrodes will respond to lower levels when the ion is in equilibrium with a complex of the ion with another species, that is, when the ion is buffered [9]. The Nernstian response of the fluoride electrode extends down to 0·5 μM if the electrode is used at low pH, where the fluoride is buffered with hydrogen ion. However, conventional reference electrodes with liquid junctions present a problem at low pH because the high ionic strength background, and the high relative mobility of hydrogen ions in the solution, lead to the generation of significant liquid junction potentials of uncertain magnitude in series with the fluoride electrode potential. However, if the reference electrode is replaced with a pH electrode, the fluoride electrode potential can be measured against the pH electrode potential in a cell without liquid junction. Under these conditions, hydrogen fluoride will be the predominant fluoride-containing species (see figure 1) [9]. A combination of a glass pH electrode and a single fluoride electrode form a differential cell, the potential difference between the electrodes being a logarithmic function of the total fluoride concentration [10, 11]. Since both the fluoride electrode and the pH glass electrode have an extremely high resistance, it is necessary to use a pH/ion-specific electrode meter containing a high impedance input in order to measure the electrical potential between the two electrodes. Equipment previously available for the assay of inorganic fluoride ion contained this kind of high impedance unit as an integral part [12–14]. However, currently available equipment does not contain such a unit [15]. This paper describes a study validating the performance of a newly developed high impedance unit used in conjunction with a standard fluoride/pH electrode combination for the measurement of serum fluoride.

### Materials and methods

### *Apparatus*

An Orion® Model 720A bench top pH/ion-specific electrode meter (Orion Research Incorporated, Cambridge, Massachusetts) was used in conjunction with an Orion® Model 94-09 Fluoride electrode, an Orion® Model 81-55 Ross-type combination pH electrode, and a newly developed high impedance converter (Sirius ASA901, Sirius Analytical Instruments Ltd, East Sussex, UK). The fluoride and pH electrodes were connected to the reference input of the 720A meter via the battery-powered, in-line high impedance converter, which converted the pH electrode output to a low impedance signal. A micro-titration cell was machined from polypropylene. This was designed to consist of a recess containing a magnetic stirring bar and a well which permitted adequate mixing of, and satisfactory electrode membrane contact, with the sample. Polypropylene flasks, beakers, micropipettes and vials were used throughout for all fluoride measurements.

### *Reagents*

Fluoride solution: 0·1 M sodium fluoride standard (Orion cat. No. 940906) was used.

*Please address all correspondence about this paper to: Dr Stephen R. Luney, 14 Huntingdale, Ballyclare BT39 9XB, UK.*

0142-0453/95 $10.00 © 1995 Taylor & Francis Ltd.

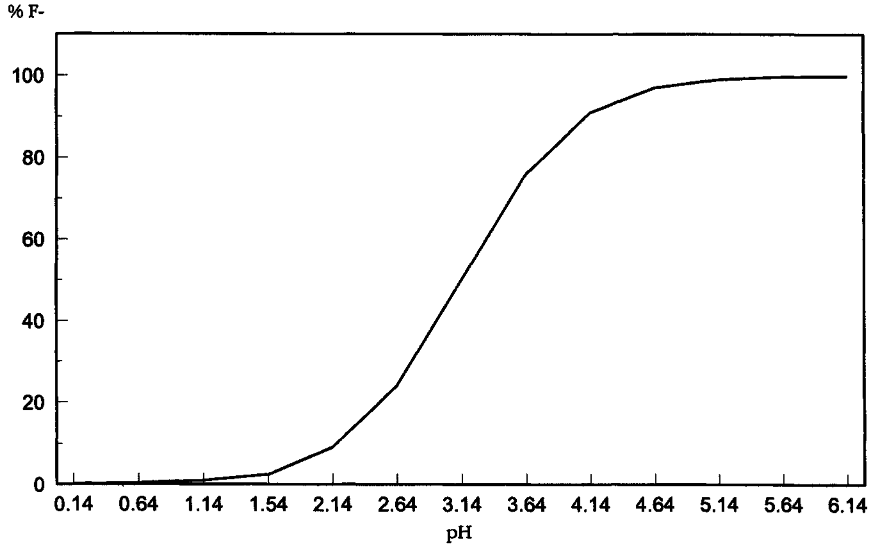

*Figure 1. Fluoride species with varying pH.*

*Fluoride analysis*

Preliminary experiments were carried out to determine the optimum pH at which serum fluoride should be measured. Fluoride solutions of concentration 0–100 μmol $l^{-1}$ were mixed with a series of solutions of 0·1, 0·2, and 0·3 N HCl (0·5 ml of solution plus 0·5 ml HCl) and the mV readings obtained by the system were recorded. The system was then calibrated using fluoride standards, 5 and 50 μmol $l^{-1}$ with 0·1 N HCl (0·5 ml standard plus 0·5 ml N HCl). Recovery in serum using 0·1 N HCl (0·5 ml serum plus 0·5 ml 0·1 N HCl, giving a resultant pH of 3·49) was measured. The recovery experiment was repeated using 0·2, and 0·3 N HCl, resulting in a pH in serum of 1·55, and 1·15 respectively.

The results of these experiments indicated that 0·2 or 0·3 N HCl could be used satisfactorily in this system. Thereafter, all experiments were carried out with 0·2 N HCl. Equilibration was achieved within one minute.

*Linearity*

Using a stock solution of sodium fluoride (1000 μmol $l^{-1}$), deionized water was spiked to give concentrations of fluoride ions from 5 to 100 μmol $l^{-1}$. This was repeated using pooled human serum in place of deionized water.

*Calibration*

A comparison of calibrations using aqueous and serum based calibrators was performed, with no statistically significant difference found between the two. The electrode was thus calibrated with two dilute fluoride standard of 5 and 50 μmol $l^{-1}$ in deionized water (0·5 ml of standard +0·5 ml 0·2 N HCl) with requirement for a change of greater than −50 mV for a ten-fold increase in fluoride ion concentration [8].

*Lower limit detection*

Lower limit of detection was determined by replicate analysis of an aqueous blank, and calculated by multiplying 2·5 times the standard deviation.

*Imprecision*

Neat serum and serum spiked with 5, 30, and 50 μmol $l^{-1}$ fluoride was assayed consecutively 10 times to give within-run imprecision. These sera were analysed on 12 occasions in three assays run on three days to calculate between-batch imprecision.

### Results

*pH range*

The response of the fluoride electrode in combination with the glass pH electrode in aqueous solutions of varying pH and fluoride concentration is shown in figure 2, which demonstrates the Nernstian response of the system over the pH range 1·15 to 3·49. The expression:

$$E_F - E_H = E_{TF} = E^\circ_{TF} - RT/F \ln[\mathrm{HF}]$$

(where $E$ is the observed e.m.f.; $E^\circ$ is the standard potential; $R$ is the gas constant; $T$ is the absolute temperature; $F$ is Faraday's constant; and $E_{TF}$ is the potential of the cell) was used to calculate the e.m.f. of the fluoride/pH electrode well as a function of fluoride ion concentration in HF solutions at different pH [10]. The calculated data are plotted in figure 3, and show a Nernstian response which becomes increasingly independent of pH as pH decreases. Because of the difficulty of measuring $E^\circ$ for the two electrodes, a value of −663·5 mV was chosen for the term $E^\circ_{TF}$ which represented the sum of the two $E^\circ$ terms. Using this arbitrary choice, the slopes and pH-dependence of the calculated curves are similar to the experimentally-determined curves. (The effect of choosing a different arbitrary value for $E^\circ_{TF}$ would be to move the curves, which would, however, remain parallel to those in figure 2.)

*Accuracy*

Over the concentration range of 2·6 to 100 μmol $l^{-1}$ fluoride in serum, recoveries using 0·1 N HCl, were

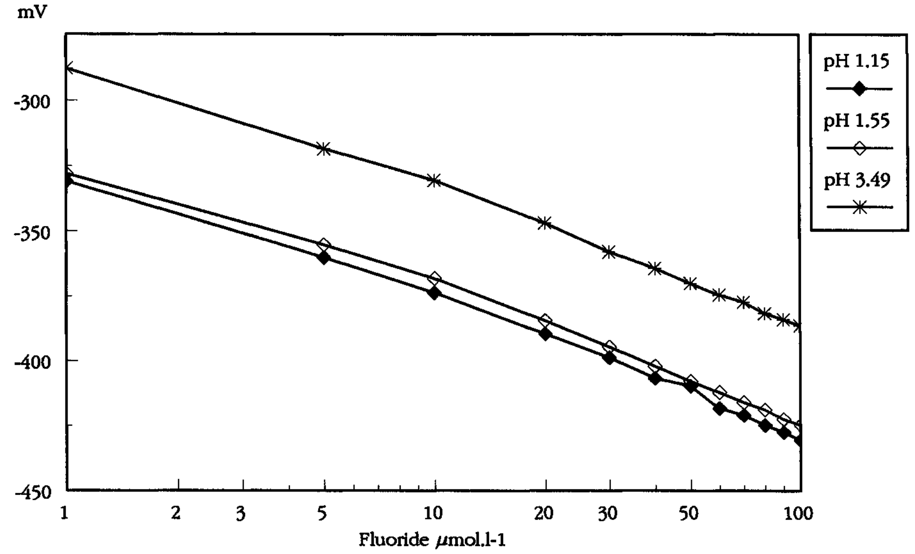

*Figure 2. Measured e.m.f. pH glass electrode/NaF, HF(aq)/fluoride ISE.*

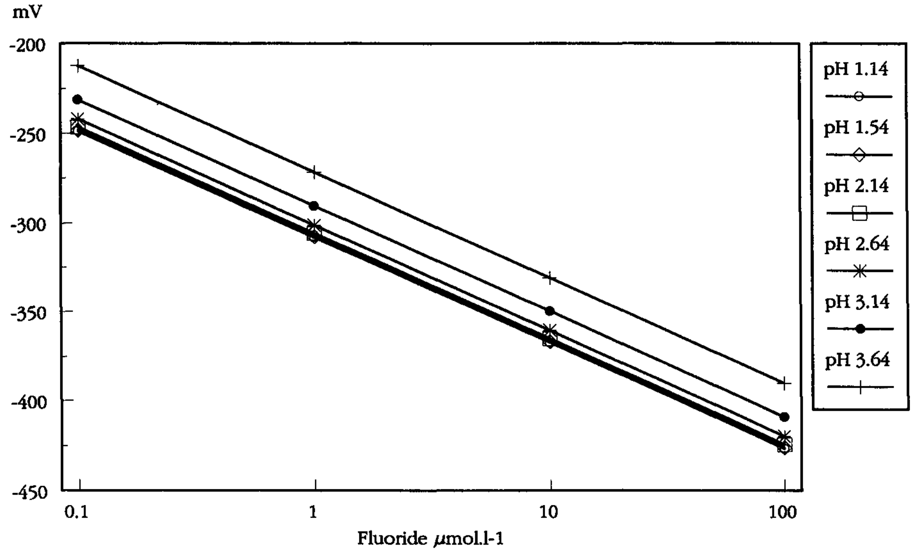

*Figure 3. Calculated e.m.f. pH glass electrode/NaF, HF(aq) fluoride ISE.*

less than 20%. In this range, serum aliquots gave recoveries in the range 94–105% using 0·2 N HCl and 0·3 N HCl.

*Linearity*

No deviation from linearity was observed in either serum or water up to a concentration of 100 µmol $l^{-1}$. The calculated slope of these two curves showed no significant difference, with values of 0·97 for water, and 0·95 for serum (see figure 4).

*Lower limit of detection*

The lower limit of detection of the method was 0·3 µmol $l^{-1}$ fluoride.

*Imprecision*

Within- and between-run imprecision is shown in table 1. Imprecision was greatest at the lowest fluoride levels, and least at the higher critical levels of fluoride concentration.

### Discussion

The combination of a new high impedance unit with the pH and fluoride electrode demonstrates a Nernstian response over a pH range of 1·15 to 3·49, with a mean slope of −56·7, over a range of 1 to 100 µmol $l^{-1}$ fluoride (see figure 2). These data are in keeping with the theoretically derived response of the system (figure 3), the curves becoming increasingly independent of pH as the pH decreases. However, recovery experiments on serum

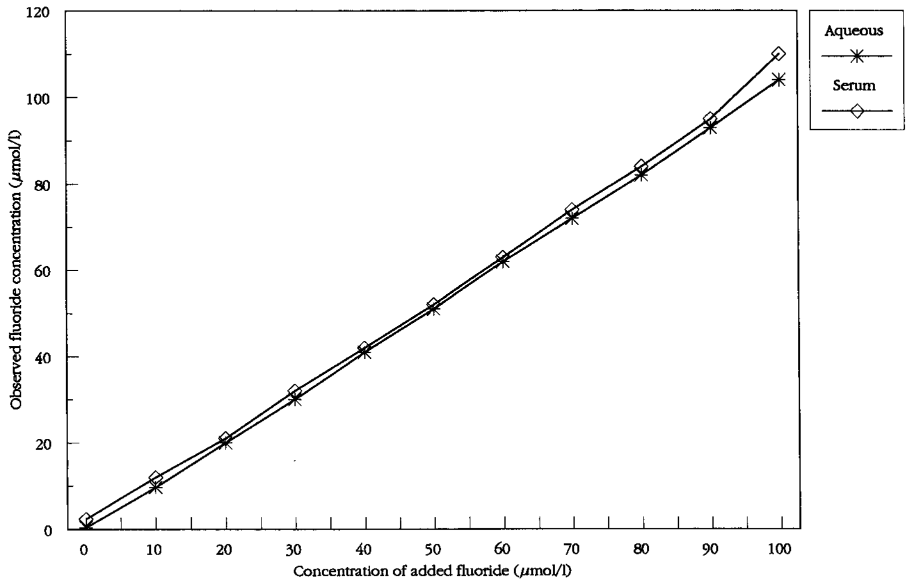

*Figure 4. Linearity of response in serum and aqueous calibrants.*

*Table 1. Imprecision of serum fluoride measurements.*

| Mean ($\mu mol\ l^{-1}$) | $N$ | Within-run CV % | Mean ($\mu mol\ l^{-1}$) | $N$ | Between-run CV % |
|---|---|---|---|---|---|
| 2·3 | 10 | 4·2 | 2·2 | 12 | 12·8 |
| 7·2 | 10 | 3·7 | 6·8 | 12 | 9·7 |
| 33·5 | 10 | 4·1 | 32·2 | 12 | 3·9 |
| 55·7 | 10 | 1·2 | 51·7 | 12 | 4·6 |

showed that only in the range pH 1·15 to 1·55 were acceptable levels of recovery found. For the purposes of the experiments, a pH of 1·55 was chosen. Mean slope observed at this pH (measured at 1·55) was −56·7 mV (CV 1·6%, $N = 9$).

At this pH, the system was found to give a linear response up to 100 $\mu mol\ l^{-1}$, and the serum and aqueous standards showed virtually the same response, as described above. Aqueous calibrants of 5 and 50 $\mu mol\ l^{-1}$ were chosen because of the clinical importance of this range. Over the first 15 to 20 minutes of use, this system demonstrates initial instability, with calibration slopes varying from −51·0 to −59·0 mV. However the system then stabilizes to give day-to-day variations in the range from −55·4 to −56·8 mV over a three-day period. These variations were reflected in greater between-batch imprecision of 12·8% at a mean level of 2·2 $\mu mol\ l^{-1}$, and 9·7% at a mean level of 6·8 $\mu mol\ l^{-1}$ than at the higher, more clinically relevant levels, where between-batch imprecision was 3·9% at a mean level of 32·2 $\mu mol\ l^{-1}$, and 4·6% at a mean level of 51·7 $\mu mol\ l^{-1}$. The low detection limit of the method of 0·3 $\mu mol\ l^{-1}$ is adequate for the levels of fluoride encountered in the general population. The recovery over the normal to pathological range of serum fluoride was acceptable. The system has a rapid equilibration time of less than one minute, an additional improvement on previous systems [9, 13].

With regard to interference by other ions, cations and most anions do not interfere with the response of the fluoride electrode to fluoride. Anions commonly associated with fluoride such as chloride, bromide, iodide, sulphate, bicarbonate, phosphate and acetate do not interfere with electrode function. The hydroxyl ion is an electrode interference, but only when the level of hydroxide is greater than one tenth that of fluoride. Some anions, such as carbonate or phosphate, make the sample more basic, increasing the hydroxide interference, but are not direct electrode interferences.

Although it is well known that ion selective electrodes are susceptible to protein build up, no deterioration in electrode performance was detected. Nonetheless, the electrode was maintained by regular cleaning with a membrane cleaning solution of 1% pepsin in 0·1 M HCl.

These data show that this new system demonstrates the accuracy, precision, and low detection limit required for the clinical measurement of inorganic fluoride in serum. The system has the potential for further development in terms of a reduction of sample size and automation. This may be of particular benefit in the evaluation of the new heavily fluorinated volatile anaesthetic agents currently undergoing development.

### References

1. Mazze, R. I., *Canadian Anaesthetic Society Journal*, **31** (1984), S16–S22.
2. Whitford, G. M. and Taves, D. R., *Anesthesiology*, **39** (1973), 416–427.

3. Mazze, R. I., Kosek, J. C., Hitt, B. A. and Love, F. V., *Journal of Pharmacology Experimental Therapeutics.* **190** (1974), 530–541.
4. Roman, R. J., Carter, J. R., North, W. C. and Kauker, M. L., *Anesthesiology*, **46** (1977), 260–264.
5. Kobayashi, Y., Ochiai, R., Takeda, J., Sekiguchi, H. and Fukushima, K., *Anesthesia and Analgesia*, **74** (1992), 753–757.
6. Jones, R. M., Koblin, D. D., Cashman, J. N., Eger II, E. I., Johnson, B. H. and Damask, M. C., *British Journal of Anaesthesiology*, **64** (1990), 482–487.
7. Kharasch, E. D. and Thummel, K., *Anesthesiology*, **75** (1991), A350.
8. Fluoride Electrode Instruction Manual, Orion Research Inc., Boston (1987).
9. Tyler, J. E. and Comer, J. E. A., *Analyst*, **110** (1985), 15–18.
10. Diggens, A. A. and Ross, J. W., UK Patent Application GB2064 131 A (1981).
11. Fry, B. W. and Taves, D. R., *Journal of Laboratory Clinical Medicine*, **75** (1970), 1020–1025.
12. Tyler, J. E., Poole, D. F. G. and Kong, K. L., *Journal of Dental Research*, **67** (1988), 677.
13. Tyler, J. E., Poole, D. F. G. and Kong, K. L., *Journal of Dentistry*, **18** (1990), 59–62.
14. Moody, G. J. and Thomas, J. D. R., *Ion-Selective Electrode Reviews*, **1** (1979), 187–206.
15. 720A Instruction Manual, Orion Research Inc., Boston.

